# An international core outcome set for primary progressive aphasia (COS‐PPA): Consensus‐based recommendations for communication interventions across research and clinical settings

**DOI:** 10.1002/alz.14362

**Published:** 2024-11-13

**Authors:** Anna Volkmer, Emily Viega Alves, Hagit Bar‐Zeev, Elena Barbieri, Petronilla Battista, Ashleigh Beales, Barbara Costa Beber, Emilie Brotherhood, Ines Ribeiro Cadorio, Maria Teresa Carthery‐Goulart, Jade Cartwright, Sebastian Crutch, Karen Croot, Maria Isabel d´Ávila Freitas, Jeanne Gallée, Stephanie M. Grasso, Katarina Haley, Heleen Hendriksen, Shalom Henderson, Lize Jiskoot, Isabel Junqueira Almeida, Jackie Kindell, Rachel Kingma, Lorinda LY Kwan‐Chen, Monica Lavoie, Adi Lifshitz‐Ben‐Basat, Regina Jokel, Aurore Mahut‐Dubos, Jordi A. Matias‐Guiu, Michèle Masson‐Trottier, Marcus Meinzer, Ellen McGowan, Carolina Mendez‐Orellana, Aaron M. Meyer, Carly Millanski, Núria Montagut, Aimee Mooney, Darby J. Morhardt, Lyndsey Nickels, Monica Norvik, Iris Edda Nowenstein, Avanthi Paplikar, Margaret Pozzebon, Antoine Renard, Leanne Ruggero, Emily Rogalski, Anna U. Rysop, Fredrik Sand Aronsson, Aida Suárez‐González, Sharon Savage, Mai Tran Thi, Kyriana Tsapkini, Cathleen Taylor‐Rubin, Donna C. Tippett, Nina Unger, Lizet van Ewijk, Sandra Wielaert, Ingvild Elisabeth Winsnes, Anne Whitworth, Ibrahim Can Yasa, David Copland, Maya L. Henry, Jason D. Warren, Rosemary Varley, Sarah J. Wallace, Chris J. D. Hardy

**Affiliations:** ^1^ Division of Psychology and Language Sciences University College London London UK; ^2^ Graduate Programme in Medical Sciences Federal University of Rio Grande do Sul Porto Alegre Brazil; ^3^ Sheba Medical Center, Tel Hashomer Ramat Gan Israel; ^4^ Mesulam Center for Cognitive Neurology and Alzheimer's Disease Northwestern University Chicago Illinois USA; ^5^ Istituti Clinici Scientifici Maugeri IRCCS Laboratory of Neuropsychology Pavia Italy; ^6^ Community Rehabilitation Unit Tasmanian Health Service Hobart Tasmania Australia; ^7^ Department of Speech, Language and Hearing Sciences Graduate Program in Rehabilitation Sciences Universidade Federal de Ciências da Saúde de Porto Alegre (UFCSPA) Porto Brazil; ^8^ Dementia Research Centre Department of Neurodegenrative Disease UCL Queen Square Institute of Neurology University College London London UK; ^9^ Center for Health Technology and Services Research (CINTESIS@RISE) Universidade Fernando Pessoa Fernando Pessoa School of Health Sciences Porto Portugal; ^10^ Human Communication, Learning, and Development Unit Faculty of Education The University of Hong Kong Hong Kong Hong Kong SAR; ^11^ Center for Mathematics Cognition and Computing Federal University of ABC Santo Andre Brazil; ^12^ Cognitive and Behavioural Neurology Unit Neurology Clinic Division Hospital das Clínicas School of Medicine University of São Paulo Butantã Brazil; ^13^ School of Health Sciences College of Health and Medicine University of Tasmania Hobart Tasmania Australia; ^14^ School of Psychology University of Sydney Sydney New South Wales Australia; ^15^ Department of Speech, Language and Hearing Sciences Federal University of Santa Catarina (UFSC) Florianópolis Santa Catarina Brazil; ^16^ Department of Medicine University of Washington Seattle Washington USA; ^17^ Departments of Speech, Language and Hearing Sciences and Neurology The University of Texas Jesse H. Jones Communication Center Austin Texas USA; ^18^ Department of Health Sciences University of North Carolina School of Medicine UNC‐Chapel Hill Chapel Hill North Carolina USA; ^19^ Alzheimer Center Amsterdam, Neurology, Vrije Universiteit Amsterdam UMC location VUmc Amsterdam The Netherlands; ^20^ Amsterdam Neuroscience, Neurodegeneration Amsterdam The Netherlands; ^21^ 18 Medical Research Council Cognition and Brain Sciences Unit University of Cambridge Cambridge UK; ^22^ Department of Neurology and Alzheimer Centre Erasmus Medical Centre Rotterdam The Netherlands; ^23^ Cognitive and Behavioural Neurology Unit Neurology Clinic Division, Hospital das Clínicas School of Medicine University of São Paulo São Paulo Brazil; ^24^ Division of Psychology, Communication & Human Neuroscience University of Manchester Manchester UK; ^25^ Speech Pathology Department Uniting War Memorial Hospital School of Psychological Sciences Macquarie University Waverley, Sydney New South Wales Australia; ^26^ Department of Special Education and Counselling The Education University of Hong Kong Hong Kong PR China; ^27^ Chaire de recherche sur les aphasies primaires progressives – Fondation de la famille Lemaire CHU de Québec – Université Laval Québec Canada; ^28^ Department of Communication Disorders Faculty of Health Sciences Ariel University Ari'el Israel; ^29^ Rotman Research Institute Temerty Faculty of Medicine University of Toronto Toronto Ontario Canada; ^30^ Lille Neuroscience & Cognition University of Lille Lille University Hospital Lille France; ^31^ Department of Neurology Hospital Clínico San Carlos San Carlos Health Research Institute (IdISSC) Madrid Spain; ^32^ Department of Neurology Johns Hopkins School of Medicine Baltimore Maryland USA; ^33^ Department of Neurology University Medicine Greifswald Greifswald Germany; ^34^ Pennine Care National Health Service Foundation Trust Manchester UK; ^35^ Health Sciences Department Speech, Language and Hearing School Faculty of Medicine Pontificia Universidad Católica de Chile Santiago Chile; ^36^ Center for Aphasia Research and Rehabilitation Georgetown University Medical Centre Washington District of Columbia USA; ^37^ Fundació de Recerca Clínic Barcelona‐IDIBAPS Barcelona Spain; ^38^ Oregon Alzheimer's Disease Research Center – Department of Neurology Oregon Health & Science University Portland Oregon USA; ^39^ Mesulam Center for Cognitive Neurology and Alzheimer's Disease Northwestern University Feinberg School of Medicine Chicago Illinois USA; ^40^ School of Psychological Sciences Macquarie University Sydney New South Wales Australia; ^41^ Department of Acquired Brain Injury Department of Education Faculty of Humanities Social Sciences, and Education UiT the Arctic University of Norway Tromsø Norway; ^42^ Speech‐Language Pathology Unit National University Hospital and Institute of Linguistics University of Iceland Reykjavik Iceland; ^43^ Department of Speech and Language Studies SpeakUp Centre for Speech Therapy & Neuro Rehabilitation Dr. S. R. Chandrasekhar Institute of Speech and Hearing Bangalore India; ^44^ Age Right Speech Pathology Melbourne Australia; ^45^ Unité PsyNcog, ULG HEC‐ULg Ecole de Gestion de l'Université de Liège Unité PsyNcog, ULG Liège Belgium; ^46^ Department of Neurology Healthy Aging & Alzheimer's Research Care (HAARC) Center University of Chicago Chicago Illinois USA; ^47^ Division of Speech and Language Pathology Department of Clinical Science, Intervention and Technology Karolinska Institutet Section of Speech and Language Pathology Medical Unit Allied Health Professionals Karolinska University Hospital Karolinska Sweden; ^48^ School of Psychological Sciences University of Newcastle Callaghan New South Wales Australia; ^49^ Department of Speech and Language Therapy Neuroscience & Cognition, Inserm UMRS1172 University of Lille Lille University Hospital Lille France; ^50^ Departments of Physical Medicine and Rehabilitation, Neurology, and Otolaryngology – Head and Neck Surgery Johns Hopkins University School of Medicine Baltimore Maryland USA; ^51^ Research Group Speech and Language Therapy Participation through Communication Research Centre Health and Sustainable Living HU University of Applied Science Utrecht Utrecht The Netherlands; ^52^ Rijndam Rehabilitation Centre Rotterdam The Netherlands; ^53^ Department of Linguistic and Scandinavian Studies University of Oslo Oslo Norway; ^54^ Department of Speech and Language Therapy Faculty of Health Sciences Bahcesehir University Beşiktaş Istanbul Turkey; ^55^ Queensland Aphasia Research Centre School of Health and Rehabilitation Sciences The University of Queensland Brisbane Australia; ^56^ Surgical Treatment and Rehabilitation Service (STARS) Education and Research Alliance The University of Queensland and Metro North Health Queensland Brisbane Queensland Australia

**Keywords:** core outcome set, dementia, interventions, outcome measures, primary progressive aphasia, speech and language therapy

## Abstract

**INTRODUCTION:**

Interventions to treat speech‐language difficulties in primary progressive aphasia (PPA) often use word accuracy as a highly comparable outcome. However, there are more constructs of importance to people with PPA that have received less attention.

**METHODS:**

Following Core Outcome Set Standards for Development Recommendations (COSSTAD), this study comprised: Stage 1 – systematic review to identify measures; Stage 2 – consensus groups to identify important outcome constructs for people with PPA (*n* = 82) and care partners (*n* = 91); Stage 3 – e‐Delphi consensus with 57 researchers.

**RESULTS:**

The systematic review identified 84 Outcome Measurement Instruments. Core outcome constructs identified included: (1) Participate in conversations with family and friends, (2) get words out, (3) be more fluent, (4) convey a message by any means, and (5) understand what others are saying. Researchers were unable to reach a consensus on measurement instruments.

**DISCUSSION:**

Further work is required to develop appropriate measurement instruments that address all core outcome constructs important to key stakeholders.

**Highlights:**

- We introduce new symptom‐led perspectives on primary progressive aphasia (PPA).
- The focus is on non‐fluent/agrammatic (nfvPPA) and semantic (svPPA) variants.
- Foregrounding of early and non‐verbal features of PPA and clinical trajectories is featured.
- We introduce a symptom‐led staging scheme for PPA.
- We propose a prototype for a functional impairment scale, the PPA Progression Planning Aid.

## 1 BACKGROUND

The term primary progressive aphasia (PPA) describes a group of language‐led neurodegenerative dementias.^1^, ^2^, ^3^ The research diagnostic criteria outline three major PPA syndromes: semantic variant (svPPA), nonfluent/agrammatic variant (nfvPPA), and logopenic variant (lvPPA).^1^ The semantic variant (svPPA) is primarily associated with frontotemporal lobar degeneration pathology (specifically TDP‐43 type C) and difficulties in word retrieval and understanding word meaning. The nonfluent/agrammatic variant (nfvPPA) is also usually associated with frontotemporal lobar degeneration pathology (most will have a primary tauopathy such as progressive supranuclear palsy or corticobasal degeneration).^4^ nfvPPA is characterized by motor planning or programming, known apraxia of speech (AOS), and/or difficulties with grammar (agrammatism). Primary progressive apraxia of speech (PPAOS) is a motor speech disorder rather than aphasia,^5^, ^6^ for the purposes of this study, and in line with consensus research diagnostic criteria,^1^ PPAOS is considered part of the broader nfvPPA syndrome. Finally, the logopenic variant (lvPPA) is commonly associated with Alzheimer's disease pathology and results in difficulties in word retrieval and phonological working memory.^3^ Frequently, people with PPA may not “fit” the standard formulations outlined in the three major PPA syndromes, presenting with mixed speech and language symptoms. These are often termed atypical, mixed, or not otherwise specified (nosPPA).^4^

Researchers from different clinical professions including speech and language therapy, psychology, neurology, neuroscience, social work, and occupational therapy have developed tailored behavioral interventions for people with PPA.^7^, ^8^, ^9^, ^10^, ^11^, ^12^, ^13^, ^14^, ^15^, ^16^, ^17^ Word retrieval training is the most studied intervention in PPA and word accuracy (its outcome measure) is a highly comparable outcome across studies.^9^ Importantly, there are more constructs, apart from word retrieval, that are important for people with PPA that have historically received less attention but have been gaining traction in recent years. Such measures are important, as people with PPA and their families have reported that measures used have not always focused on the outcomes that are important to them.^18^

Although outcome measurement instruments require alignment with intervention targets, a core outcome set (COS) has the potential to improve comparability by standardizing the measurement and reporting in intervention studies related to a particular health condition.^19^ A COS facilitates comparison across intervention trials, has the potential to allow for grouping of data, and, by involving key stakeholders in the process of developing COSs, ensures intervention research results are more relevant to them.^20^ Previous work has aimed to develop a COS for use in the evaluation of non‐pharmacological interventions for all‐cause dementia, which identified communication as a core outcome construct likely to be valued highly by people living with dementia.^21^, ^22^ Communication was not, however, defined beyond “Being able to communicate with others.” However, identifying specific measures for this broad construct was also beyond the scope of that research.^22^ Work has also been undertaken to identify a COS for post‐stroke aphasia. The Research Outcome Measurement in Aphasia ROMA‐COS identified five essential outcome constructs and appropriate measurement instruments that address each domain^23^, ^24^ including: language, communication, patient‐reported satisfaction with treatment methods impact and methods, emotional wellbeing, and quality of life. However, PPA presents unique challenges in comparison to post‐stroke aphasia, both due to its progressive nature and to its significant and evolving issues with nonverbal cognition, behavior, and motor abilities.^25^, ^26^, ^27^

The aim of this international cross‐disciplinary collaboration was, therefore, to identify outcome constructs that are important to people with PPA and their care partners and explore relevant outcome measurement instruments for researchers and clinicians working in the field of PPA interventions. The long‐term goal is to identify “what” constructs and “how” best to measure these constructs as outcomes, will inform future developments in PPA intervention research, as well as have the potential to improve the relevance of research to end‐users. This, in turn, will benefit people with PPA and their care partners by increasing access to evidence‐based interventions that address outcomes that are important to them.

## 2 METHODS

The objectives of this study were:

1. To describe how speech, language, and communication outcomes have been measured in PPA intervention studies to date.
2. To identify the most important outcome constructs of communication intervention from the perspectives of people with PPA and their care partners.
3. To determine if there is multi‐disciplinary consensus on a COS for communication interventions for PPA (the COS‐PPA) using existing measures for use in research and clinical settings.

### 2.1 Scope

The health condition and population covered by this COS:

1. The population is people living with PPA and their care partners, as defined by the current diagnostic criteria^1^ (NB: For the purposes of the current study, people with PPAOS have been captured within the nfvPPA group).

The interventions covered by this COS:

2 The COS‐PPA may be used to measure the outcomes of communication interventions for people living with PPA and their care partners (behavioral, pharmacological, or neuromodulation). This includes all impairment, activity, and participation interventions as defined by The International Classification of Functioning, Disability, and Health.^28^

**RESEARCH IN CONTEXT**

1. **Systematic review**: Following Core Outcome Set Standards for Development Recommendations (COSSTAD), this study comprised three stages: Stage 1, The systematic review identified 84 Outcome Measurement Instruments. Stage 2, Core outcome constructs identified included: (1) Participate in conversations with family and friends, (2) get words out, (3) be more fluent, (4) convey a message by any means, and (5) understand what others are saying. Stage 3. Researchers were unable to reach a consensus on measurement instruments.
2. **Interpretation**: The initial identification of the first consensus‐based recommendations for a core outcome set for PPA has the potential to ensure that PPA intervention research can produce more comparable and generalizable results in the future. Further work is required to develop appropriate measurement instruments that address all core outcome constructs important to key stakeholders.
3. **Future directions**: This research study has developed the first consensus‐based recommendations for a core outcome set for PPA – the COS‐PPA – aiming for widespread use within research on interventions for people living with PPA and their care partners. We advocate that this core outcome set be used alongside, rather than instead of, study‐specific measures that are more aligned with the intervention targets.

The settings covered by this COS:

3 The settings covered by this COS‐PPA are intervention research and clinical delivery of interventions to people with PPA and their care partners. The geographical setting encompasses most major World Health Organization regions,^29^ where researchers and health care professionals work with people with PPA and their care partners in a variety of languages.

This study followed the COS Standards for Development Recommendations (COSSTAD)^20^ and the results of the COS‐PPA are reported in line with the 18‐item Core Outcome Set‐STAndards for Reporting (COS‐STAR) (see Supplementary information for a completed COS‐STAR checklist). The COS‐PPA development protocol has been published in a peer‐reviewed scientific journal.^30^ The COS‐PPA was registered on the COMET website in March 2021.^31^ In addition, Stage 1 systematic review was preregistered on PROSPERO in December 2022: CRD42022367565.

### 2.2 Ethical approval

All aspects of the study were conducted according to the Declaration of Helsinki.

The Stage 2 UK collaboration was undertaken as part of the Rare Dementia Support (RDS) Impact Study which received approval from the UCL Research Ethics Committee (8545/004: Rare Dementia Support Impact Study). All consent sessions were video recorded, in line with the approved procedure outlined in the RDS Impact study protocol (Brotherhood et al., 2020). For the collaboration with Dr. Carolina Mendez in Chile, ethical approval was granted by the Pontificia Universidad Catolica de Chile Ethics Committee, ID no. 190510002. For the collaboration with Dr. Regina Jokel in Canada, ethical approval was granted by Baycrest, Research Ethics Board REB 22–37. For the collaboration with Dr. Jade Cartwright and Dr. Cathy Taylor‐Rubin in Australia, ethical approval was granted by Southeastern Sydney Local Health District HREC 2022/ETH02740. For the collaboration with Dr. Iris Nowenstein in Iceland, ethical approval was granted by the Ethics Committee of the National University Hospital of Iceland (1/2024). For the collaboration with Dr. Avanthi Paplikar in India, ethical approval was granted by the Bangalore Speech and Hearing Research Foundation. For the collaboration with Prof Marcus Meinzer, Anna U. Rysop, and Nina Unger in Germany, ethical approval was granted by the Greifswald University Ethics Committee, Germany, Reference BB 130/22. For the collaboration with De Ines Cadorio in Portugal, ethical approval was granted by Ethics Committee of the University Fernando Pessoa Prot n. 50/C.E > del 28/02/22. For the collaboration with Dr. Petronilla Battista in Italy, ethical approval was granted by the Ethics Committee of the IRCCS Giovanni Paolo II Bari, Prot. n. 80/CE Maugeri on 17/02/2022. For the collaboration with Dr. Adi Lifshitz‐Ben‐Basat and Hagit Bar‐Zeev in Israel, ethical approval was granted by the Ariel University Ethics Board, Israel. For the collaboration with Dr. Maya Henry and Carly Millanski in America, ethical approval was given by the Office of Research Support and Compliance and the University of Texas at Austin's Institutional Review Board IRB ID STUDY00000717‐MOD06. For the collaboration with Dr. Lizet van Ewijk, Dr. Sandra Wielaert, Dr. Lize Jiskoot, Janna van Egmond, Heleen Hendriksen, and Antoinette Keulen in the Netherlands, ethical approval was granted by Amsterdam UMC under the number 2023.0098. For the collaboration with Dr. Monica Norvik in Norway, ethical approval was granted by the Norwegian Agency for Shared Services in Education and Research (SIKT) ref: 865145. For collaboration with Maria Isabel d'Avila Freitas in Brazil, ethical approval was granted by the Hospital of Clinics – Faculty of Medicine – University of Sao Paulo (USP) ref: 4.142.664.

Stage 3 work was granted ethical approval by the Chairs of UCL Language and Cognition Department Ethics, Project ID LCD‐2023–06. All participants involved were asked to complete an online written consent form before participation in the e‐Delphi survey.

### 2.3 Design

**FIGURE 1:**
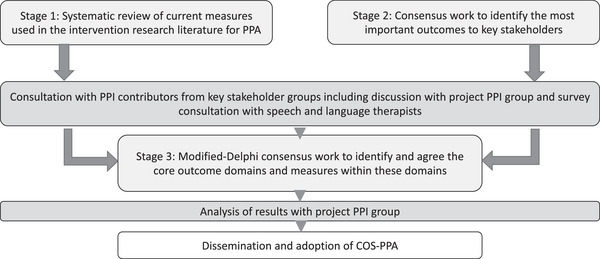
Workflow for core outcome set primary progressive aphasia (COS‐PPA).

The COS‐PPA was developed over three stages: Stage 1, a systematic review of outcome measurement instruments described in the intervention research literature for PPA; Stage 2, consensus groups with people with PPA and their care partners to identify the most important outcome constructs for them; and Stage 3, a modified e‐Delphi consensus study with researchers working in the field of PPA intervention research to agree to the core outcome constructs and relevant measurement instruments. Consultation with the project Patient and Public Involvement (PPI) advisory group informed the refinement of the work to develop the COS‐PPA (as outlined in the COS‐PPA protocol paper^30^). This approach follows the COMET handbook,^32^ a guide for developing COSs. Figure 1 provides an overview of the workflow for the COS‐PPA study.

#### 2.3.1 Stage 1: A systematic review of outcome measurement instruments used in the intervention research literature for PPA

The aim of this systematic review was to examine the speech, language, and communication measures used in research studies exploring interventions for PPA (behavioral, pharmacological, or neuromodulation) to date to:

1. Identify the constructs that are measured by these outcome measurement instruments, and how they align with the constructs identified in Stage 2 consensus groups, and inform Stage 3 agreement regarding relevant measurement instruments.
2. Identify the PPA variants each measure has been used with and the languages in which the measure is available.

##### Procedures

This systematic review replicated and updated a recent review undertaken in the field of PPA interventions,^13^ employing the same search strategy but expanding this to include pharmacological interventions. The review update was undertaken on March 31 2023. Studies were included that: (a) described original research (any design); (b) were published in a peer‐reviewed journal (exclusive of conference abstracts); (c) investigated behavioral, pharmacological, or neuromodulation treatment for speech and/or language; (d) were conducted with one or more people with a diagnosis of PPA; and (e) reported treatment outcomes for at least one individual. Nine databases were searched: Medline, CINAHL (all via EBSCOhost), Embase, PsycInfo, ComDisDome, Scopus, and Web of Science, including non‐English studies. Titles and abstracts of identified articles were reviewed by the lead author (A.V.) and independently reviewed by a second author (C.J.D.H.) and assessed for inclusion or exclusion. All articles that were included then underwent a second round of full‐text screening independently by A.V. and C.J.D.H. Any discrepancies in ratings were discussed until an agreement was reached.

Data extraction and analysis: All publicly available or published primary and secondary outcome measurement instruments of speech, language, and communication reported in the final list of studies were extracted and documented in a spreadsheet by AV. The data had already been collected for papers identified by Wauters et al.,^13^ and further data were only sought if the description of measures used did not include a specific tool. Each measure was considered in terms of the constructs it examined, in line with the World Health Organization‐International Classification of Functioning, Disability and Health (WHO‐ICF) framework and how these aligned with the constructs identified by participants in Stage 2 consensus work. Data were then extracted from each article with regard to which PPA variant the measures were used (lvPPA, svPPA, nfvPPA, and nosPPA). Finally, one author (A.V.) explored which language each measure was available in and for which population the measure had been developed. Where possible, psychometric data on each measure were also extracted. The full protocol for this review was registered on PROSPERO in December 2022: CRD42022367565.

#### 2.3.2 Stage 2: Consensus work – Focus groups using a nominal group technique to identify the most important outcome constructs for people with PPA and their care partners

The important intervention outcome constructs for people with PPA and their family members were identified using group consensus methods. A nominal group technique (NGT) protocol previously developed to meet the needs of people with stroke aphasia by one of the authors (S.J.W.),^33^ was modified for people with PPA.

##### Recruitment

Fifteen international research sites were established. Site leads (authors C.M., R.J., J.C., CT.‐R., A.P., M.M., A.U.R., N.U., I.C., P.B., A.L.B.B., H.B.Z., M.H., C.M., S.W., L.J., J.W., H.H., A.K., L.V., M.N., I.E.W., A.R., M.I.D.F., I.C.Y, and E.B.) were identified through international networks including the collaboration of aphasia trialists (CATs), the International PPA SLT/P network and through attendance at international conferences. A procedure manual and slide deck were shared with collaborators to ensure methodological consistency across sites. Materials were translated as required, and country‐specific ethical approvals were obtained as per local requirements.

##### Procedures

Collaborators approached people with PPA, and their care partners, using their local networks, and invited them, via email, to participate in a focus group meeting. Participants completed consent procedures as required by each collaborator's institution. Collaborators collated demographic data from participants on age, sex, type of PPA, and time since diagnosis. Meetings were held either online, via video conferencing, or in person, depending on the local COVID‐19 restrictions and ethical committee guidance at the time. Participants with PPA and care partners attended separate group meetings. People with PPA and care partners were able to participate independently; thus, not all care partners were the actual care partners of the participants with PPA who participated and vice versa. Participants with PPA were asked, “What would you most like to change about your communication and the way PPA affects your life?” Care partners were asked, “What would you most like to change about your family member's communication and the way PPA affects your life?” Following NGT^34^, ^35^ methods, each participant in a group was independently invited to generate a list of items in response to the question. These were then shared one by one with the remaining group members until no more new ideas were generated. Having collated a list of ideas across the group, each participant was asked to identify their individual top three items in order of priority. A copy of the study manual, which also outlines communication support strategies provided during the process, is available with the protocol article.^30^

##### Analysis

Demographic data were anonymized, and mean values were calculated for each country. The site leads (collaborator in each country) weighted answers from each person with PPA or each care partner (items ranked first were weighted with 3, second with 2, and third with 1), and then aggregated individual scores to produce a final list of results, identifying the top three ranked items for each group (people with PPA and their care partners) in each country. Anonymized results (the top three items) collected from different countries were shared with the lead author (A.V.). By weighting each country's responses in line with the NGT methodology^34^, ^35^ (items ranked first weighted with 3, second with 2, and third with 1) every country's results were given equal weight, regardless of how many participants. These results were then aggregated to produce a prioritized list, representing data from across all contributing countries.

#### 2.3.3 Stage 3: An international e‐Delphi exercise to gain consensus on the COS‐PPA

To generate a final COS‐PPA, a modified e‐Delphi consensus process was undertaken with a team of international cross‐disciplinary collaborators.^34^ This included identifying and voting on the core outcome constructs and measurement instruments for each key construct identified in Stages 1 and 2.

##### Participants

Researchers were invited by email, to participate in the e‐Delphi exercise if they: (a) had participated in Stage 2 NGT work, and/or (b) were researchers and authors of studies identified in Stage 1 systematic review, and/or (c) were contacts established via networks including CATs, the International SLT/P PPA network and the International Society of Frontotemporal Dementias (ISFTD).

##### Procedures

Emails were sent inviting researchers to participate in the study. If they agreed by return of email, they were sent a link to complete an online consent form and brief survey collecting demographic information (including affiliation, professional background, research interests, country of current work, qualification, number of PPA participants seen as part of research, languages in which research had been undertaken) and availability to attend a group meeting hosted on a video conferencing platform (Zoom).

Prior to attending video conferencing meetings, researcher participants were asked to complete an online vote rating the importance of all outcome constructs identified in Stage 2 of the study. Researcher participants rated each construct on a scale of importance with 9 being the most important and 1 being the least important. Rankings of 7–9 indicated critical importance, 4–6 indicated outcomes that were considered important but not critical, while ratings of 1–3 were outcomes considered of limited importance using the Grading of Recommendations Assessment, Development, and Evaluations (GRADE) scale.^36^ Participants were also invited to put forward additional constructs that they felt were important, but that had not been included. A mean ranking was generated based on all respondents’ ratings. Constructs were also given ratings based on the prioritization by the two key stakeholder groups in Stage 2, such that the top‐ranked item from Stage 2 received a rating of 9, the next a rating of 8, and so on to 1. This resulted in every construct receiving three ratings (one from people with PPA, one from care partners, and one from researchers). Subsequently, a mean rating was calculated across PwPPA, care partners, and researcher participants. Constructs that received an overall rating of 6–9 progressed to the next stage of voting.

Research participants who had completed the online ratings were invited to participate in one of three 60‐min online meetings, hosted on Zoom and recorded for later checking of data collection if required. In these meetings, results from the ranking of all constructs were presented. For the constructs that progressed to the next stage of voting, participants were provided with the names of the outcome measurement instruments that had been identified in Stage 1 systematic review. Only those outcome measurement instruments that were reported as measuring the agreed constructs were presented. Research participants were given a brief description of each outcome measurement instrument, including the languages in which it was available and the population for which it was developed. Participants were then asked to vote whether each outcome measurement instrument, in their opinion, measured the corresponding construct. They were encouraged to make additional suggestions of relevant measures for each construct or add any relevant comments. Following the group meetings, results were aggregated. Measures that did not receive any votes were excluded from the final round of voting.

In the third and final round of voting, research participants who had attended the Zoom meetings were emailed a survey link and asked to vote on which measure they felt best measured the core outcome construct in question. Research participants were provided with results from the previous round of voting and information on each measure. The information included a link to access measures where they were freely available or a link to a reference describing the measure. Where possible, references were included relating to psychometric properties of measures and data relevant to PPA. Languages in which each tool was available and approximate administration time were also provided. Participants were also able to indicate if they did not feel there was currently an appropriate measure available.

##### Analysis

Demographic information was summarized using descriptive statistics. Anonymized voting data collected in the modified e‐Delphi consensus study were aggregated.[Fig alz14362-fig-0002]

## 3 RESULTS

### 3.1 Stage 1: Systematic review of current measures in the research literature on interventions for PPA

**FIGURE 2 alz14362-fig-0002:**
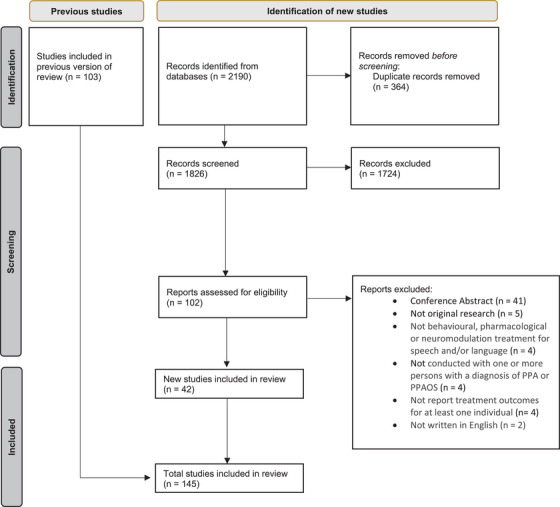
PRISMA flow diagram for core outcome set primary progressive aphasia (COS‐PPA) review.

The update of Wauters et al.’s^13^ systematic review, employing the same search strategy but expanding this to include neurostimulation and pharmacological studies, identified 1826 papers. There was 96.8% interrater reliability by A.V. and C.J.D.H. in the review of titles and abstracts of identified articles. The remaining 3.23% of papers (59) were discussed and agreed by consensus. In addition to the 103 papers identified by Wauters et al., another 102 possible papers were considered for inclusion. Independent full‐text screening achieved 85% interrater agreement and full agreement was reached after discussion. Forty‐two papers of the 102 additional papers were identified for inclusion, resulting in a total of 145 papers included in the final review. The PRISMA diagram in Figure 2 details the reasons for exclusion. For a full list of papers and risk of bias evaluation, please see Volkmer et al.^37^

#### 3.1.1 Data extraction

A final list of 84 measures was extracted. Of these, 5 were specifically developed for people with PPA, and 20 for people with dementia (inclusive of PPA). Thirty‐four measures were available in English only. Of the 84 measures, 42 were described by the authors as measuring a construct that potentially aligned with the finalized constructs in Stage 3 Delphi‐consensus task and consequently were presented for voting. Table 1 lists the 84 measures and indicates those that were included in the consensus voting. A full analysis of the extracted data is reported elsewhere.^37^

**TABLE 1:** List of measures, their reference, and related data extracted from review.

	Measure name	In what languages is the tool available?	Construct (synthesized by lead author A.V. based on authors descriptions of how the tool has been used in PPA intervention research)	Which population was the tool developed for?
1.	Batteria per l'analisi dei deficit afasici – BADA^38^	Italian	Get the words out	Stroke aphasia
2.	Boston Naming Test ‐BNT^39^	English, Icelandic, Spanish, Turkish, Finnish, Hebrew, Korean, Chinese, Brazilian Portuguese, Italian, Dutch, Greek, Norwegian	Get the words out	Stroke aphasia
3.	Cookie Theft^40^	English, Icelandic, Swedish, Hindi, Arabic, Spanish, Norwegian	Get the words out	Adults with communication difficulties
4.	Hopkins Assessment of Naming Actions – HANA^41^	English	Get the words out	Stroke Aphasia and Primary Progressive Aphasia
5.	Isaac Set Test^42^	English	Get the words out	Dementia
6.	Northwestern Assessment of Verbs and Sentences – NAVS^43^	English, Italian	Getting words out	Adults with acquired neurological diseases
7.	Object and Action Naming Battery – OANB^44^	English, Greek, Spanish,	Getting words out	Adults with acquired language impairments
8.	Oral Denomination 80 – DO‐80 Naming Test^45^	French	Get the words out	Aphasia
9.	Philadelphia Naming Test short – PNT^46^	English	Get the words out	Stroke aphasia
10.	Picture Confrontation Oral Naming – PNT80^47^	English	Get the words out	Stroke aphasia
11.	Rapid Automatized Naming Test – RAN^48^	English	Getting words out	Adults/Children
12.	Revised‐English‐Hebrew Aphasia Battery^49^	Bilingual/English‐Hebrew	Get the words out	Stroke aphasia
13.	Snodgrass and Vanderwart Picture Naming Test^50^	English, Spanish, Russian, Icelandic, Chinese, Italian, Portuguese/Brazilian Portuguese, Tamil, French, Croatian, Persian	Get the words out	Adults with no communication difficulties
14.	Test of Adolescent/Adult Word Finding – TAAWF^51^	English	Get the words out	Adolescents and adults
15.	Verbal Fluency Test^52^	14 languages	Get the words out	Adults without communication difficulties
16.	Persian Aphasia Test^53^	Iranian	Get the words out, understanding	Stroke aphasia
17.	Western Aphasia Battery – WAB‐R^54^	31 language including English	Get the words out, understanding	Stroke aphasia
18.	Comprehensive Aphasia Test – CAT^55^	Available in 14 languages and being translated into further 6 at time of writing.	Get the words out, understanding	Stroke aphasia
19.	Aachen Aphasia Test – AAT^56^	German, English, Portuguese, Italian, Turkish, Dutch	Get the words out, understanding	Stroke aphasia
20.	Psycholinguistic Assessment in Chinese Aphasia – PACA^57^	Chinese	Get the words out, understanding	PPA
21.	Screening for Aphasia in NeuroDegeneration – SAND^58^	Italian	Get the words out, understanding	PPA
22.	Test of language development‐2^59^	English	Get the words out, fluency & understanding	Children with language difficulties
23.	Psycholinguistic Assessments of Language Processing in Aphasia – PALPA^60^	16 languages including English	Get the words out, understanding	Stroke Aphasia
24.	Alzheimer's Disease Assessment Scale – Cognitive Subscale – ADAS‐cog^61^	107 languages including English (original language)	Behavior, get the words out, understanding	Alzheimer's disease
25.	Mini‐Mental State Examination – MMSE^62^	15 languages including English	Get the words out, understanding	Dementia
26.	Porch Index of comm ability^63^	English	Get the words out, understanding	Stroke Aphasia
27.	Token test^64^	English, Russian, Indonesian, Chinese, Moroccan, Italian, Spanish, Brazilian Portuguese, Dutch, Norwegian	Understanding	Stroke aphasia
28.	Test for the Reception of Grammar‐ TROG and TROG‐2^65^, ^66^	Multiple languages including English, French, Brazilian Portuguese, Deutsche, Tamil, Norwegian	Understanding	Developed for children with communication difficulties
29.	Apraxia Battery for Adults^67^	English, Greek, Icelandic, Korean	Fluency	Stroke apraxia
30.	Diagnostic Instrument for Apraxia of Speech^68^	Dutch, English	Fluency	Stroke
31.	American Speech‐Language‐Hearing Association Functional Assessment of Communication Skills for Adults – ASHA FACS^69^	English, Chinese, Italian, Brazilian, Portuguese	Convey a message by any means, Participate in conversations with family and friends	Adults and children with communication difficulties
32.	Communication Activities of Daily Living, Second Edition – CADL‐2^70^	English, Spanish, Icelandic, Italian, Arabic	Convey a message by any means	stroke, traumatic brain injury, dementia, primary progressive aphasia
33.	Texas Functional Living Scale^71^	English	To go out independently, convey a message by any means	Alzheimer's disease and Elderly
34.	The Speech Questionnaire^72^	English, French	Convey a message by any means	Stroke Aphasia
35.	ACOM‐ Aphasia Communication Outcome Measure^73^	English	Participate in conversations with family and friends	Stroke aphasia
36.	American Speech‐ Language‐Hearing Association Quality of Communication Life Scale – ASHA QoCL Scale^74^	English	Participate in conversations with family and friends	Adults with communication difficulties
37.	American Speech–Language–Hearing Association Functional Communication Measures – ASHA‐FCM^75^	English	Convey a message by any means, Participate in conversations with family and friends	Adults with communication difficulties
38.	Assessment of activities of daily living and instrumental activities of daily living – ADL/IDL^76^	English	Participate in conversations with family and friends	Alzheimer's disease
39.	Aphasia Impact Questionnaire – AIQ‐21^77^	English, Norwegian	Participate in conversations with family and friends	Stroke Aphasia
40.	Communication Confidence Rating Scale in Aphasia^78^	English‐ only at present	Participate in conversations with family and friends	Stroke Aphasia
41.	Communicative Effectiveness Index – CETI^79^	English, Icelandic, Norwegian, Dutch	Participate in conversations with family and friends	Stroke Aphasia
42.	Modified ASHA Quality of Communication Life Scale^80^	English	Participate in conversations with family and friends	Adults with communication impairments
43.	Neuropsychiatric Inventory – NPI^81^	Translated into approximately 40 languages including English	Behavior and personality	Dementia
44.	Frontal Behavioral Inventory – FBI^82^	English, Italian, Brazilian Portuguese, Norwegian	Behavior and personality	bvFTD
45.	Frontal Assessment Battery – FAB^83^	13 languages including English.	Behavior and disease progression	Frontotemporal dementia
46.	Clinical Dementia Rating Scale‐ Sum of Boxes – CDR‐SOB Executive interview^84^	Available in multiple languages including English, Spanish, French, German, Dutch, Italian, Japanese, Korean, Chinese, and Portuguese	Disease progression	Alzheimer's disease
47.	Global Deterioration Scale – GDS^85^	English	Disease progression	Dementia
48.	Unified Parkinson's Disease Rating Scale – UPDRS^86^	27 languages including English	Disease progression	Parkinson's disease
49.	The Montreal Cognitive Assessment – MoCA^87^	36 languages including English	Disease progression.	Dementia
50.	Patient Health Questionnaire^88^	Arabic, English, French, Italian, German, Hindi, Japanese, Korean, Chinese – simple and traditional, Thai	Feeling positive	Adults with mood disorders
51.	Generalized Anxiety Disorder assessment‐GAD^89^	60+ languages including English	Feeling positive	Adults with anxiety
52.	The Positive and Negative Affect Scale – PANAS^90^	English, Japanese, Spanish	Feeling positive	Adults
53.	Burden of Stroke Scale^91^	English	Feeling positive	Stroke Aphasia
54.	Stroke and Aphasia Quality of Life Scale‐39 – SAQOL‐39^92^	19 languages including English	Feeling positive	Stroke aphasia
55.	Stroke Aphasia Depression Questionnaire – SADQ^93^	English	Feeling positive	Stroke aphasia
56.	Clinical Global Impression‐Improvement^94^	English	Feeling positive	Adults with depression
57.	HDRS‐ Hamilton Depression Rating Scale^95^	English, Turkish, Lebanese, Persian, Greek, African languages	Feeling positive	Adults with depression
58.	Hamilton Anxiety Scale^96^	English, Cantonese for China, French,. and Spanish	Feeling positive	Anxiety
59.	Patient Health Questionnaire‐9 – PHQ‐9^97^	49 languages including English	Feeling positive	Depression
60.	Hopkins Verbal Learning Test^98^	English, Spanish, Greek, Persian	Word recall	Dementia, older adults
61.	Pyramids and Palm Trees Test – PPTT^99^	English, Brazilian Portuguese, Dutch, Norwegian, French	Object knowledge	Stroke aphasia
62.	Northwestern Anagram Test – NAT ^100^	English, Italian, German	Construction of sentences	PPA
63.	Kissing and Dancing Test – KDT^101^	English, Brazilian‐Portuguese	Understanding words	bvFTD
64.	Northwestern Assessment of Verb Inflection – NAVI^102^	English, Persian	Comprehension and production of action verbs	Adults with acquired neurological diseases
65.	John Hopkins University Dysgraphia Battery^103^	English	Writing	Stroke aphasia
66.	Rey Auditory Verbal Learning Test – RAVLT^104^	Seven languages including English, Icelandic	Verbal memory	Alzheimer's disease
67.	Birmingham Object Recognition Battery – BORB^105^	–	Object recognition	Neuropsychological disorders
68.	Gray Oral Reading Test‐4^106^	English	Reading	Children
69.	User Experience Questionnaire^107^	30 languages	Experience of speech therapy	Adults
70.	Test of Nonverbal Intelligence‐3 – TONI‐3^108^	Nonverbal	Non‐verbal problem solving	Children and adolescents
71.	Comprehensive Test of Nonverbal Intelligence‐2 ‐CTONI‐2^109^	Nonverbal	Non‐verbal problem solving	Children and adults
72.	Wisconsin Card Sorting Test – WCST^110^	–	Abstract reasoning	Children and adults
73.	Assessment for Living with Aphasia^111^	English	Quality of life	Stroke Aphasia
74.	Trail Making Test^112^	Several languages including English, Brazilian Portuguese, Italian, Dutch, Spanish, Norwegian	Cognition	Adults
75.	The Stroop Test^113^	English, Chinese, Brazilian Portuguese, Italian, Norwegian	Cognition	Adults
76.	Colored Progressive Matrix – CPM^114^	–	Cognition	Children and less able adults
77.	Cognitive Linguistic Quick Test – CLQT^115^	English, Chinese	Cognition	Aphasia
78.	Wechsler Adult Intelligence Scale fourth edition – WAIS IV^116^	English, Spanish, Italian, Dutch, Norwegian	Cognitive ability	Adults without communication difficulties
79.	D‐KEFS‐ Delis‐Kaplan Executive Function System Assessment^117^	English, Norwegian	Cognition	Adults and children
80.	Mattis Dementia Rating Scale^118^	English, Spanish, French, Brazilian Portuguese	Cognition	Dementia
81.	The Crossing Off Test‐ COT‐1^119^	–	Visual cognition	Adults
82.	Communicative Activity Log – CAL^120^	English, Korean, Persian	Participation in daily life	Stroke aphasia
83.	Alzheimer's Disease Cooperative Study – Activities of Daily Living Scale^121^	English	Participation in daily life.	Alzheimer's disease
84.	Bayer Activities of Daily Living scale^122^	Spanish, Korean, German, Portuguese, and English	Participation in daily life.	Dementia

*Note*: Measures highlighted in gray represent those that were included in Stage 3 Delphi consensus study.

### 3.2 Stage 2: Consensus work – Focus groups using a nominal group technique to identify the most important outcome constructs for people with PPA and their care partners

#### 3.2.1 Participants

The 15 collaborating countries that participated in Stage 2 NGT study comprised Brazil, Canada, Chile, France, Germany, Italy, India, Israel, Netherlands, Norway, Portugal, Spain, Turkey, the United Kingdom, and the United States of America.

Across these countries, 82 participants with PPA took part in the NGT study, of whom 46% were male (*n* = 38). Participants with PPA represented all three major variants: 27% svPPA (*n* = 22), 30% lvPPA (*n* = 25), and 23% nfvPPA (*n* = 19), though several participants did not have a subtype diagnosis (20%, *n* = 16). The mean age of the people with PPA was 69 years (SD = 5.4), and participants had a mean average of 32 months (SD = 16.7) post‐diagnosis. Ninety‐one care partners (NB: care partners were separately recruited and did not necessarily represent the partners of the participants in the PPA groups) took part in the study, of whom 37% were male (*n* = 34). Care partners also represented each of the major PPA variants; 25% svPPA (*n* = 23), 32% lvPPA (*n* = 29), 22% nfvPPA (*n* = 20), and participants with no subtype diagnosis (21%, *n* = 19). The mean age of care partners was 62 years (SD = 9.5), and the average number of participants was 29 months (SD = 19.4) post‐diagnosis. Table 2 provides an overview of participant demographics per participating country.

**TABLE 2:** Participant demographics for nominal group consensus process.

	People with PPA								Care partners							
Country	*n*	Mean age (years)	Male:female ratio	svPPA	lvPPA	nfvPPA	nosPPA	Mean time since onset (months)	*n*	Mean age (years)	Male:female ratio	svPPA	lvPPA	nfvPPA	nosPPA	Mean time since onset (months)
Turkey	6	57	2:4	3	2	1	0	8	6	40	1:5	3	2	1	0	8
Spain	6	64	2:4	3	3	0	0	36	6	58	3:3	3	3	0	0	36
Israel	3	76	3:0	0	0	0	3	60	3	71	0:3	0	0	0	3	60
UK	5	68	3:2	0	3	2	0	30	5	61	2:3	1	4	1	0	31
France	4	68	1:3	2	1	1	0	27	4	68	1:3	2	1	1	0	26
Chile	15	70	6:9	2	5	1	7	26	21	70	8:13	3	7	1	10	20
Portugal	2	72	2:0	1	0	0	1	7	2	71	0:2	1	0	0	1	7
Italy	7	69	3:4	1	3	3	0	32	7	51	3:4	1	3	3	0	3
Canada	3	75	0:3	1	2	0	0	63	4	64	2:2	0	4	0	0	65
Netherlands	10	68	5:5	2	2	5	1	24	10	63	5:5	2	2	5	1	24
Brazil	3	73	1:2	0	1	1	1	23	3	61	1:3	0	1	1	1	2
Germany	5	72	2:3	2	0	1	2	41	4	60	1:3	0	0	1	3	40
Norway	2	74	1:1	1	0	0	1	31	2	76	1:1	1	0	0	1	31
USA	6	60	3:3	1	3	2	0	45	5	60	1:4	1	2	2	0	49
India	5	66	4:1	3	0	2	0	30	9	53	5:4	5	0	4	0	30
Total:	82	69 (SD 5.4)	38:44	22	25	19	16	32 (SD 16.7)	91	62 (SD 9.5)	34:58	23	29	20	19	29 (SD 19.4)

Abbreviations: lvPPA, logopenic variant PPA; nfvPPA, nonfluent agrammatic PPA; nosPPA, not otherwise specified; PPA, primary progressive aphasia; SD, standard deviation (no standard deviations are available for individual countries); svPPA, semantic variant PPA; USA, United States of America; UK, United Kingdom.

#### 3.2.2 Stage 2: Identification of outcome constructs

Results from the aggregation of the top three ranked outcome constructs for people with PPA resulted in a total list of 13 items across all participating countries. The most highly ranked outcome was *to be able to get the words out*, the second was *to talk with people*, and the third was *to be more fluent*. The complete list of the 13 generated outcome constructs is given in Table 3.

**TABLE 3:** Ranking of core outcome constructs for people with PPA and their care partners, aggregated across 15 participating countries.

Order of ranking	People with PPA	Care partners
First	1. Get the words out	1. Get the words out
Second	2. Talk with people	2. Have conversations with family and friends
Third	3. Be more fluent	3. Convey a message by any means 4. Speak fluently
Fourth	4. To understand and follow conversation 5. Tell people how to talk with me	5. Talk about the future
Fifth	6. Convey a message by any means	6. Deal with changes in behavior
Sixth	7. Not get worse	7. Talk about sensitive issues 8. Reduce frustration 9. Access to SLT 10. Get more information about PPA and the stages of the disease
Seventh	8. To be respected/increased awareness	11. For CP to know how to help 12. That the person understands 13. Understanding of PPA in the wider community/environment 14. Be more confident 15. Be less dependent
Eighth	9. Have more SLT	16. Joy 17. To be partners, not carer and cared for
Ninth	10. Use the phone	
Tenth	11. A cure 12. Go out shopping on my own	
Eleventh	13. Know others like me	

Abbreviations: CP, care partner; PPA, primary progressive aphasia; SLT, Speech and Language Therapy.

The aggregation of the top three ranked outcome constructs for care partners resulted in a total list of 17 items across all participating countries. The most highly ranked outcome was identical to people with PPA: *to be able to get the words out*, followed by *to have conversations with family and friends*. Next were two outcomes that received equal ranking: *conveying a message by any means* and *speaking fluently*. The complete list of 17 constructs is shown in Table 3.

### 3.3 Stage 3: Consensus work to identify and agree on core outcome constructs and measurement instruments

#### 3.3.1 Participants

Fifty‐seven of the 84 researchers who were approached agreed to participate in Stage 3 consensus work. These researchers all completed the initial survey, providing an overview of their demographics and rating the importance of the outcome constructs. Eight participants were unable to attend any of the video conferencing meetings due to sickness or other commitments. Thirty‐nine of the 57 respondents were speech and language therapy researchers, 13 were psychology researchers, one neurologist, and seven were from other professional backgrounds. They represented 17 countries, with 38 respondents reporting they delivered interventions in languages other than English, whereas 38 reported delivering interventions in English. Table 4 provides a detailed summary of their demographic information.

**TABLE 4:** Demographic details of the Stage 3 participants.

Demographic characteristics	Respondent (*n* = 57)
Professional background	
Speech and language therapist	39
Psychology	13
Neurology	1
Other (e.g., social work)	4
Research interests (multiple answers permitted)	
Speech, language, and communication interventions	53
Interventions with care partner	38
Psychological/counseling	24
Neuromodulation interventions	15
Other (arts, palliative and advance care interventions)	5
Pharmacological interventions	2
Country of work	
Australia	7
Brazil	3
Canada	4
Chile	1
France	3
Germany	3
HK	2
Iceland	1
India	1
Israel	1
Italy	1
Norway	2
Portugal	1
Spain	2
Sweden	2
Turkey	1
UK	7
USA	15
Treating language (multiple answers permitted)	
Chinese	1
English	38
French	6
German	4
Hebrew	1
Icelandic	1
Italian	3
Norwegian	2
Portuguese	5
Polish	1
Russian	1
Spanish	6
Swedish	2
Turkish	1
Other – not specified	4
No. of people with PPA ever recruited to research	
1–5	6
6–20	17
21–50	11
51–100	11
>100	12

Abbreviations: HK, Hong Kong; PPA, primary progressive aphasia; UK, United Kingdom; USA, United States of America.

#### 3.3.2 Consensus work to agree on core outcome constructs

The total list of 25 outcome constructs identified from the aggregated Stage 2 NGT data was presented for rating by the 57 researchers. Mean ratings of importance across researcher participants are presented in Table 5. Aggregated and overall mean ratings from participants with PPA, their care partners and the researchers are also presented in Table 5. Overall, two constructs, “to be able to participate in conversations with family and friends” and “to be able to get the words out” were rated as the most important outcomes. “To be more fluent” was rated as the next most important, followed by “to be able to convey a message by any means” and “to understand what others are saying in conversation.” The next two constructs focused on emotional‐behavioral outcomes: “to be able to talk about sensitive issues” and “for family members to understand how to deal with changes in behavior.” After these, the next two constructs focused on societal and service level outcomes: “to increase awareness of speech and language therapy among the public” and “to have more speech and language therapy.” The full list of constructs and their ratings is listed in Table 5. Constructs that were voted as equally important by two participant groups are also presented in Table 5. The total list was presented to the PPI advisory group who recommended that the first five constructs (rating 5–9) progress to Stage 3, as they felt they comprised a group of speech, language, and communication outcome constructs that should be treated together and, distinct from lower‐rated emotional‐behavioral and societal and service level outcomes. An analysis of outcome constructs for individual participating countries in relation to cultural differences is reported elsewhere.^123^

**TABLE 5:** Outcome constructs listed in order of importance based on total mean rating across PwPPA, CPs, and researcher participants.

Construct	PwPPA (converted from ranking in Stage 2)	CP (converted from ranking in Stage 2)	Researchers (mean rating across researcher participants)	Total	Mean rating across PwPPA, CPs, and researcher participants
To be able to talk and participate in conversations with family and friends^a^	8	8	8	24	8
To be able to get the words out^a^	9	9	6	24	8
To be more fluent^a^	7	7	6	20	7
To be able to convey a message by any means^a^	5	7	7	19	6
To understand what others are saying in conversation	6	3	7	16	5
To talk about sensitive issues	0	6	7	13	4
For family members to understand how to deal with changes in behavior	0	5	7	12	4
To increase awareness and understanding of PPA among the public^a^	3	3	6	12	4
To have more speech and language therapy	2	4	6	12	4
To understand more about PPA and what to expect in the future	0	4	7	11	4
To maintain relationship between a person with PPA and their families and friends	0	2	8	10	3
For people around the person to know how to help in conversation	0	3	7	10	3
To be less frustrated	0	4	6	10	3
To halt the progress of the disease	4	0	6	10	3
To feel positive	0	2	6	8	3
To feel included	0	0	7	7	2
To meet other people affected by PPA	1	0	6	7	2
To feel supported	0	0	7	7	2
To feel hopeful	0	0	7	7	2
To be able to go out independently	0	0	6	6	2
To be more confident	0	0	6	6	2
To be less anxious	0	0	6	6	2
To be more accepting of the diagnosis of PPA	0	0	6	6	2
To improve speech clarity	0	0	5	5	2
To be able to use the telephone	0	0	5	5	2

*Note*: Including illustration of overlap of equally rated construct ratings across people with PPA, care partners, and researchers

Abbreviations: CP, care partner; PPA, primary progressive aphasia; PwPPA, person with PPA.

^a^ Constructs that were voted as equally important by two participant groups.

#### 3.3.3 Consensus work to agree on core outcome measurement instruments

Forty‐nine researchers participated in work to identify a set of measures for the first five rated outcome constructs. In the first round of voting, measures identified in Stage 1 systematic review were presented under each construct and researchers were asked to vote on whether these tools measured the construct in question. They were also invited to suggest additional tools and make additional written comments. Any measure that received a vote in this round, as well as any additional suggested measures, were taken through to the final round of voting. In this final round, participant researchers were contacted via email to complete an online vote. They were asked to select the measure they felt best measured the construct or were able to indicate that they did not feel they could agree on a measure at this current time. Several respondents had indicated in the first round of voting that some constructs should be split, therefore respondents were also able to vote on this.

The American Speech‐Language‐Hearing Association Functional Assessment of Communication Skills for Adults (ASHA FACS)^69^ received the most votes (*n* = 20, 41%) for the construct *to be able to participate in conversation with family and friends*. Thirteen researchers (27%) felt they could not currently identify a measure for this construct, with no measure receiving more than 50% agreement. Nineteen researchers (39%) voted that the construct *to be able to get words out* should be split into confrontation naming and connected speech. The Cookie Theft picture description task^40^ received the next most votes under this construct (*n* = 9, 18%). More than half of the researchers (*n* = 31, 63%) voted that *to be more fluent* should be split into motor speech and connected speech, with The Cookie Theft,^40^ Verbal Fluency Test^52^ and measures of words/min coming in second with an equal (small) number of votes each (*n* = 4, 8% each). The ASHA FACS^69^ also received the most votes (*n* = 24, 49%), albeit not enough to reach consensus, for the construct *to convey a message by any means*, with 9 (18%) researchers voting they could not currently identify a measure. Finally, the sentence comprehension subtest from the Comprehensive Aphasia Test (CAT)^55^ was voted for by 27 researchers (49%) under the construct *to be able to understand what others are saying in conversation*, with 7 (14%) researchers rating that they felt they could not currently identify a measure. The final set of measures voted for under each construct is listed in Table 6 below.

**TABLE 6:** Top five outcome constructs with sum of votes for identified outcome measurement instruments (*n* = 49).

Construct	Outcome measurement instruments, or alternative options (cannot identify any measure/construct needs to be split) listed in order of votes from highest to lowest					
*To be able to participate in conversations with family and friends*	ASHA FACS (*n* = 20)	Cannot agree a measure at present (*n* = 13)	ASHA QCL (*n* = 4) SAQOL (*n* = 4)	CCRSA (*n* = 3) AIQ (*n* = 3)	ACOM (*n* = 1) ALA (*n* = 1)	
*To be able to get words out*	This construct needs to be split into confrontation naming and connected speech (*n* = 19)	The Cookie Theft (*n* = 9)	Connected speech measure‐ story retell (*n* = 5)	Verbal Fluency Test (4)	BNT (*n* = 3) CAT‐ naming subtest (*n* = 3) Cannot agree measure (*n* = 3)	AAT (*n* = 1) MLSE (*n* = 1) (NB: One participant did not vote on this construct)
*To be more fluent*	This construct needs to be split into motor speech and connected speech (*n* = 31)	The Cookie Theft (4) Verbal Fluency Test (4) Measure of words p/min (4) Cannot agree measure (4)	ABA (2)			
*To convey a message by any means*	ASHA FACS (24)	Cannot agree a measure at present (11)	Scenario Test (8)	ADL/IDL (3)	ANELT (2)	Social Networks Scale (1)
*To understand what others are saying*	Sentence comprehension subtest on CAT (27)	Cannot agree a measure (7)	Sentence comprehension subtest on PALPA (4) Sentence Comprehension subtest on WAB (4)	AIQ (3)	TROG (2)	Token test (1) SAND (1)

Abbreviations: AAT, Aachen Aphasia Test^56^; ABA, Apraxia Battery for Adults^68^; ACOM, Aphasia Communication Outcome Measure^73^; ADL/IDL, Assessment of activities of daily living and Instrumental activities of daily living^76^; AIQ, Aphasia Impact Questionnaire^77^; ALA, Assessment for Living with Aphasia^111^; ANELT, Amsterdam‐Nijmegen Everyday Language Test^124^; ASHA FACS, American Speech‐Language‐Hearing Association Functional Assessment of Communication Skills for Adults^69^, ^125^; ASHA QCL, ASHA Quality of communication life scale^80^; BNT, Boston Naming Test^39^; CAT, Comprehensive Aphasia Test^55^; CCRSA, Communication Confidence Rating Scale in Aphasia^78^; MLSE, Mini Linguistic State Exam^126^; PALPA, Psycholinguistic assessments of language processing in aphasia^60^; SAQOL, Stroke and aphasia quality of life scale‐39^92^; SAND, Screening for Aphasia in NeuroDegeneration^58^; TROG, Test for Reception of Grammar^65^, ^66^; WAB, Western Aphasia Battery.^54^

### 3.4 Summary of results

This study describes the development of the first consensus‐based recommendations for a COS for communication interventions in PPA using existing measures. The Stage 1 systematic review update demonstrates the considerable heterogeneity in outcome measurement instruments used in the PPA intervention research literature to date. In Stage 2, 82 people with PPA and 91 care partners were recruited across 15 different countries, representing one of the largest studies in the field of outcome measures for communication interventions in PPA. Constructs identified as important were relatively similar across people with PPA and their care partners, with a focus on getting words out, having conversations, fluency, and conveying a message by any means. Finally, Stage 3 e‐Delphi consensus study brought together 57 multidisciplinary researchers spanning 17 countries, who voted on the constructs identified in Stage 2 to prioritize the top five outcome constructs: (1) *Participate in conversations with family and friends*, (2) *get words out*, (3) *be more fluent*, (4) *convey a message by any means*, and (5) *understand what others are saying*. Although there was no measure on which even a majority of researchers could agree, two measures received the highest number of votes: the ASHA FACS^69^ for the constructs (1) *participating in conversation with family and friends* and (2) *conveying a message by any means*; and the sentence comprehension subtest from the CAT^55^ for (5) *understanding what people say*.

## 4 DISCUSSION

This study highlights the lack of suitable and publicly available outcome measurement instruments that are informed by the needs of people with PPA. Of the 84 published or publicly available measures only five had been developed for people with PPA (Psycholinguistic Assessment in Chinese Aphasia – PACA^57^; Northwestern Anagram Test – NAT^100^; Screening for Aphasia in NeuroDegeneration – SAND^58^; Hopkins Assessment of Naming Actions^98^; Communication Activities of Daily Living – CADL‐2^70^), yet none of these aligned with the five outcome constructs. Only the SAND^57^ was identified as a potential candidate, but given it is only available in one language (Italian) likely contributed to its lack of votes in the final round of the e‐Delphi study. Of the two identified measurement instruments, the sentence comprehension subtest for the CAT^55^ has helpfully been translated into 20 languages. The CAT has been shown to have good‐to‐excellent inter‐rater reliability, and concurrent validity indicates that the sentence comprehension subtest correlates with another sentence comprehension test (Test for Reception of Grammar (TROG^65^) at 0.885). The ASHA FACS^69^ is only available in English and European Portuguese but has been reported as a reliable instrument with an inter‐rater reliability for communication independence in stroke aphasia and, external validity data demonstrates correlations between ASHA‐FACS and the Western Aphasia Battery^54^ of > 0.5, and the Functional Independence Measures of > 0.61.^125^ However, both CAT and ASHA‐FACS are designed for people with post‐stroke aphasia, a condition characterized by acute onset and a potentially improving, rather than a progressive deteriorating trajectory. In addition, while several measurement instruments (20) identified in Stage 1 review had been designed specifically with the needs of people with dementia in mind, none were considered aligned with the final COS outcome constructs. It is likely that the unique communication difficulties experienced by people with PPA are captured less by measures for people with generic cognitive decline, than those designed for people with specific language difficulties. In fact, several researchers voted that they did not feel there was a suitable outcome measurement instrument available to address the outcome constructs identified in this COS‐PPA. This highlights the urgent need for more measures that are designed for people with PPA and their care partners.

People with PPA and their care partners identified several speech, language, and communication outcome constructs that traversed specific linguistic capabilities related to language impairments associated with PPA. Interestingly, the top prioritized item, *having conversations with family and friends*, is similar to the dementia COS, where communication and relationships were identified as the top outcome construct.^21^, ^22^ The COS‐PPA, however provides more specificity, by distinguishing between communication activities such as *having a conversation with family and friends* and *conveying a message by any means*. Despite this, two of the constructs identified by people with PPA and their care partners in this COS‐PPA, *thinking of words* and *being more fluent*, were somewhat ambiguous, resulting in researchers being unable to agree on a relevant measure. *Thinking of words* and *being more fluent* evoke the experiences of trying to convey information to others but could also refer to naming objects or articulatory accuracy. Alternatively, rather than the constructs themselves lacking specificity, it is possible that the issue may reflect the heterogeneity of available assessments for these constructs. Although this highlights the limitations of trying to identify a “catch all” COS for all three PPA variants, it could be argued that people do not necessarily experience their disease as a discrete set of symptoms but instead experience their disease through the lens of everyday interaction.^127^ Despite this desire for specificity, future work to develop new measures may benefit from being grounded in the language people with PPA and their care partners use to describe their experiences.

Another important result of the current endeavor is the potential clinical implications of the constructs that were identified as important for people with PPA and their care partners. These outcomes point to the need to revisit traditional clinical priorities that have often supported more impairment‐based, over or before more compensatory approaches. Previous research has highlighted the need to take a person‐centered approach when working with people with PPA.^128^, ^129^ When people are unable to identify their own goals because they are unclear about the role of the speech and language therapist,^25^ offering people with PPA and their care partners a range of possible intervention goals based on the outcome constructs identified in this study may facilitate this process.

Given that researchers from a range of disciplines participated in Stage 3 e‐Delphi consensus study, our results highlight a shared view that outcome measurement is both important and challenging across intervention research for PPA. The participating researchers reported conducting a range of psychological, speech and language, social, pharmacological, and neuromodulation intervention research. As a result, these researchers viewed outcome constructs and measurement instruments from several different theoretical and experiential perspectives (ICF, WHO).^28^ Consequently, the final COS may be more or less distal from the interventions they investigate. For example, a peer support intervention for people with PPA is quite distal from the person's ability to understand what other people are saying. Therefore, an outcome measurement instrument related to understanding may not be relevant, as the intervention might not be expected to impact this construct. A peer support intervention might instead impact participation in conversation or conveying a message by any means, and COS measures related to these constructs may be more relevant. We believe that it is important that a COS should be used carefully. They should be used to complement measures that are more proximal to the intervention and that may be specific to that intervention. We also suggest that, in future iterations of a COS PPA, there is a need for a larger pool of outcome measurement instruments that also address constructs such as well‐being and coping.

### 4.1 Strengths and limitations

This international collaboration was influenced by a desire to conduct compatible research across languages and cultures. To this end, this study has several strengths in its breadth and reach, with representation from all World Health Regions (WHO),^29^ except for Africa. Consequently, there remains a limited understanding of how clinicians work in non‐western regions, and what their needs are, and what the needs of their patients with PPA are. Translation of Stage 2 stimulus question into different languages, and consequent translation of respondents’ responses back into English enabled increased participation across different countries. However, this also means that there was a risk that the respondents' intent may have been changed or biased in the process. To reduce this risk, the lead researcher met with each relevant collaborator to discuss and agree on the meanings of the questions and constructs during translation. It is also important to highlight that the respondents in Stage 2 consensus groups predominantly represented people with PPA in the early‐to‐middle stages of the disease, highlighting the issue common across the PPA research literature, that there is little exploration of the severe and palliative stages of the condition. As a consequence of the different professional and language backgrounds of researchers in Stage 3, people were not always familiar with the full list of measurement instruments they were being asked to rate. Not all measurement instruments were freely available, but in the final round of voting and where available, links to the measures and relevant psychometric data were provided for participant researchers to access, should they wish. Whereas the consensus process’ used in Stages 2 & 3 ensured anonymity, it might have been valuable to explore different demographic variables such as PPA variant and time since diagnosis for people with PPA and care partners, and research interests or professional backgrounds among researchers. This study aimed to identify outcome constructs across PPA variants; however, further consideration of constructs in relation to different PPA variants and times since symptoms onset may be particularly valuable in the development interventions targeting symptoms associated with specific PPA variants or stages of progression.

## 5 FUTURE RESEARCH

This research study has developed the first consensus‐based recommendations for a COS for PPA – the COS‐PPA – aiming for widespread use within research on interventions for people living with PPA and their care partners. We advocate that this COS be used alongside, rather than instead of, study‐specific measures that are more aligned with the intervention targets. Given that this COS focused on the needs of people with PPA and their care partners across the world, using the COS‐PPA can help demonstrate how well interventions address these identified needs. The use of the COS‐PPA should lead to the potential for future systematic reviews/meta‐analyses to combine and compare data sets and thereby inform clinical decision‐making about the most effective interventions for people with PPA. This study has highlighted the need for more PPA relevant measures, including Patient Reported Outcome Measures, and the need to ensure the adaptation for a range of languages and cultures. Additionally, further consensus work to expand the COS‐PPA to include measures of well‐being and coping is essential to capture the breadth of the needs of people with PPA and their care partners. This study has started a conversation across the research discipline about how best to measure outcomes for people with PPA and their care partners. Ongoing revision of a COS will be needed as our understanding of PPA evolves and additional measures are developed, particularly for languages other than English. These measures may have relevance for people living with non‐language led dementias, who also have speech, language, and communication needs.^130^

## AUTHOR CONTRIBUTIONS

A.V. is the chief investigator, she conceived the study, led the proposal and protocol development, and led all three stages of the research, analysis, and manuscript preparation. C.J.D.H. contributed to the study design, development of the proposal, and co‐led stage 1 of the research. S.W. and D.C. contributed to the study design. R.V., M.H., and J.D.W. contributed to finalizing the proposal. C.M., R.J., J.C., C.T.‐R., A.P., M.M., A.U.R., N.U., I.C., P.B., A.L.B.B., H.B.Z., M.H., C.M., S.W., L.J., J.W., H.H., A.K., L.V., M.N., I.E.W., A.R., M.I.D.F., I.C.Y., and E.B. conducted research for Stage 2 and participated in stage 3 of the study. E.B., P.B., A.B., B.C.B., E.B., I.R.C., M.T.C.‐G., J.C., S.C., K.C., L.V.E., Mid.F., J.G., S.M.G., K.H., S.H., J.K., L.L.Y.K.‐C., R.K., M.L., AL.‐B.‐B., R.J., A.M.‐.D, J.A.M.‐G., M.M.‐T., M.M., E.M., C.M.‐O., A.M.M., N.M., A.M., C.M., D.J.M., L.N., M.N., I.E.N., A.P., M.P., A.R., L.R., E.R., A.U.R., F.S.A., A.S.‐G., S.S., T.M.T., K.T., C.T.‐R., D.C.T., N.U., I.E.W., A.W., S.W., I.C.Y., and M.L.H. participated in Stage 3 of the study. All authors have contributed to manuscript preparation.

## CONFLICT OF INTEREST STATEMENT

The authors declare no conflicts of interest. Author disclosures are available in the supporting information.

## CONSENT STATEMENT

All human subjects provided informed consent.

## Supporting information



Supporting Information
